# Cellular projections from sensory hair cells form polarity-specific scaffolds during synaptogenesis

**DOI:** 10.1101/gad.259838.115

**Published:** 2015-05-15

**Authors:** Eliot Dow, Kimberly Siletti, Albert J. Hudspeth

**Affiliations:** Howard Hughes Medical Institute, Laboratory of Sensory Neuroscience, The Rockefeller University, New York, New York 10065, USA

**Keywords:** auditory system, filopodium, lateral line, ribbon synapse, vestibular system, zebrafish

## Abstract

The assembly of a nervous system requires the extension of axons and dendrites to specific regions where they are matched with appropriate synaptic targets. Although the cues that guide long-range outgrowth have been characterized extensively, additional mechanisms are required to explain short-range guidance in neural development. Using a complementary combination of time-lapse imaging by fluorescence confocal microscopy and serial block-face electron microscopy, we identified a novel type of presynaptic projection that participates in the assembly of the vertebrate nervous system. Synapse formation by each hair cell of the zebrafish's lateral line occurs during a particular interval after the cell's birth. During the same period, projections emerge from the cellular soma, extending toward a specific subpopulation of mature hair cells and interacting with polarity-specific afferent nerve terminals. The terminals then extend along the projections to reach appropriately matched presynaptic sites, after which the projections recede. Our results suggest that presynaptic projections act as transient scaffolds for short-range partner matching, a mechanism that may occur elsewhere in the nervous system.

## Abstract (precis)

Using a complementary combination of time-lapse imaging by fluorescence confocal microscopy and serial block-face electron microscopy, Dow et al. identified a novel type of presynaptic projection that participates in the assembly of the vertebrate nervous system.

Like many other aquatic vertebrates, zebrafish possess lateral line organs that sense water currents (Metcalfe et al. 1985). The posterior lateral line on each side of a 3-d-old larva initially comprises seven neuromasts, each of which is a compact cluster of mechanosensitive hair cells separated by supporting cells and surrounded by mantle cells (Ghysen and Dambly-Chaudiere 2004). Hair cells occur in two oppositely polarized subpopulations: Half of them are sensitive to mechanical stimulation in the anterior direction, and half of them are responsive in the posterior direction (Flock and Wersäll 1962; López-Schier et al. 2004). New hair cells arise throughout a fish's life by mitosis of precursor cells, each of which yields one anteriorly and one posteriorly polarized hair cell (López-Schier and Hudspeth 2006). Two hours after mitosis, the daughter cells undergo a characteristic rearrangement of their somata (Wibowo et al. 2011; Mirkovic´ et al. 2012) before passing through early, intermediate, and late stages of differentiation, defined by their morphology and physiology (Wibowo et al. 2011; Kindt et al. 2012). By 18 h after mitosis, the hair cells are functionally mature (Fig. 1F). From the posterior lateral line ganglion, which lies immediately caudal to the ear, several bipolar neurons extend sensory axons to contact a neuromast (Metcalfe et al. 1985). Each afferent fiber receives synaptic input from hair cells of only one polarity, a specificity that occurs independently of hair cell activity (Nagiel et al. 2008, 2009). To learn how this synaptic pattern arises, we studied synapse formation by newly differentiated hair cells in larvae 2–4 d post-fertilization (dpf).

**Figure 1.**
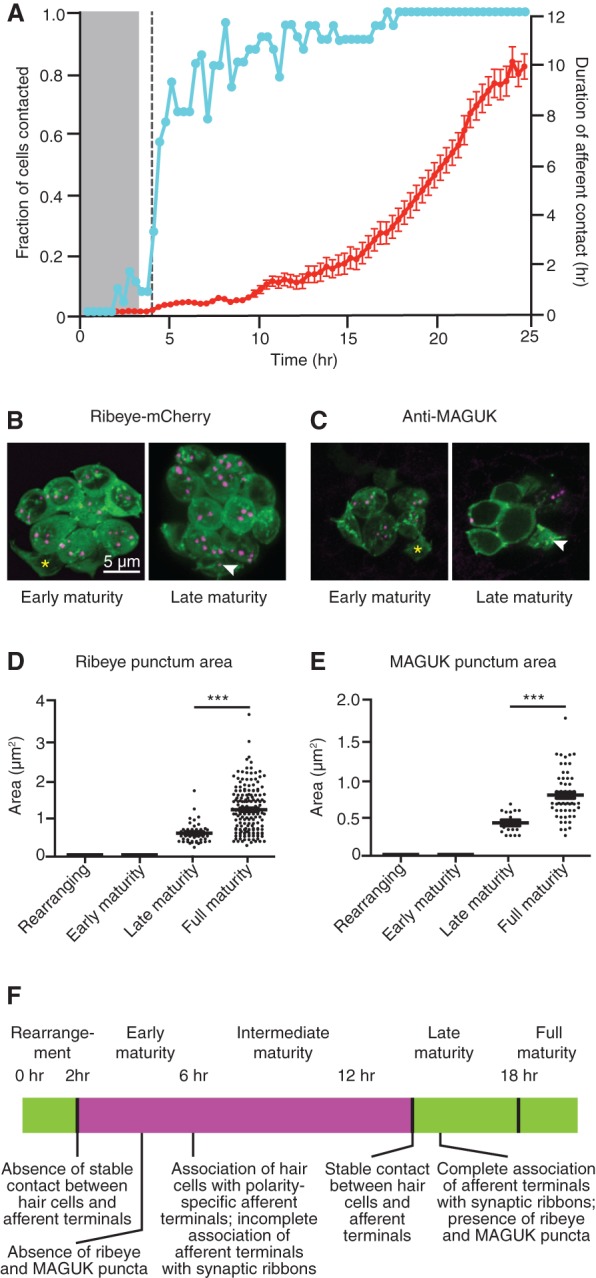
Hair cells form synapses 2–15 h after mitosis. (*A*) The fraction of nascent hair cells making contact with afferent terminals (blue) rises briskly following cellular rearrangement. Although the contacts are transitory at the outset, their duration increases progressively until, by 15 h after mitosis, the slope of the line approaches unity, indicating complete stability (red). Time is denoted in hours after mitosis. The gray band marks the period of cellular rearrangement, and the vertical dashed line marks the emergence of projections. *N* = 30. (*B*–*E*) Ribeye-mCherry fusion protein (*B*,*D*) and membrane-associated guanylate kinase (MAGUK) (*C*,*E*) puncta (arrowheads) appear in late maturity and full maturity hair cells (magenta) but are absent from rearranging cells and early maturity cells (asterisks in *B*,*C*). For *D* and *E*, *P* < 0.0001; for *D*, *N* = 8, 34, 47, and 176 measurements for the respective hair cell stages; for *E*, *N* = 8, 16, 16, and 58 measurements. (*F*) A time line of hair cell differentiation. The magenta box denotes the extension of projections from nascent hair cells from shortly after their rearrangement until stable synapses have formed (see Figs. 2–4).

## Results

We first sought to determine when differentiating hair cells form synapses. By using fluorescence confocal microscopy to observe newly arisen hair cells and afferent fibers during the 20 h following mitosis, we found that hair cells made minimal contacts with axons during rearrangement and completed the process without stably associating with afferent terminals. Immediately following rearrangement, however, contacts between hair cells and afferents increased significantly, and, by 15 h after mitosis, hair cells had associated stably with terminals (Fig. 1A). We additionally confirmed that the presynaptic and postsynaptic markers of functional ribbon synapses, ribeye and membrane-associated guanylate kinase (MAGUK) (Sheets et al. 2011), were absent from hair cells at early maturity but appeared by late maturity (Fig. 1B–F).

Although confocal microscopy provided a useful means of assessing the period of synaptogenesis, we were unable to identify the functional polarities of the axonal terminals by that means. In order to determine whether hair cells make polarity-specific synapses from the outset, we investigated three-dimensional reconstructions of neuromasts by serial block-face electron microscopy (SBEM) (Supplemental Fig. S1; Supplemental Movie 1; Denk and Horstmann 2004). A neuronal arbor was classified as an afferent terminal if it was juxtaposed to one or more hair cell synaptic ribbons. In contrast, an efferent terminal made extensive contact with all hair cells in a neuromast without apposition to any synaptic ribbons. An afferent terminal's functional polarity was assigned on the basis of the polarity of the mature hair cells to whose synaptic ribbons it was juxtaposed, which could be determined by the orientation of the relevant hair bundles.

We found that by early maturity, only half (six of 12) of the synaptic ribbons in hair cells were juxtaposed with afferent nerve fibers, indicating that synapse formation continued during this period. By late maturity, nearly all synaptic ribbons (21 of 22) were juxtaposed with afferent terminals. At all stages of differentiation, the afferent terminals at synaptic ribbons represented the polarity-appropriate subpopulation (early maturity, six of six; late maturity, 20 of 21). These results indicate that synapses form 2–15 h after mitosis and that polarity specificity commences early in this process.

**Figure 2.**
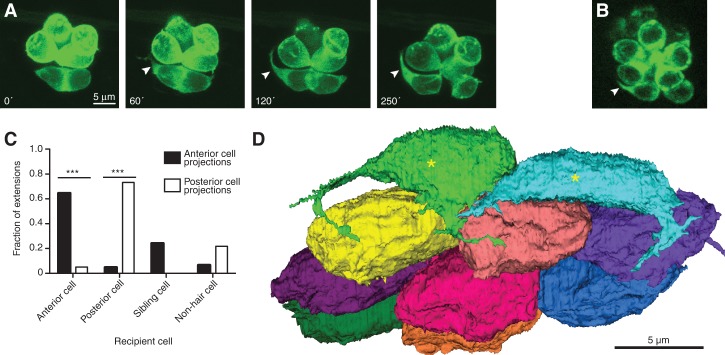
Nascent hair cells extend dynamic basal projections. (*A*) Time-lapse imaging by fluorescence confocal microscopy of hair cells expressing GFP fused to actin shows the emergence of a basal projection (arrowheads) shortly after the completion of rearrangement. Times are denoted in minutes from the end of rearrangement. (*B*) A hair cell projection (arrowhead) contains the Map1b-GFP fusion protein (green), indicative of the presence of microtubules. The scale accords with that in *A*. (*C*) Projections extend predominantly toward mature hair cells of the same polarity and occasionally toward a sibling cell or a nonhair cell. *P* < 0.0001 for both group comparisons; *N* = 9 for anteriorly polarized hair cells, and *N* = 12 for posteriorly polarized cells. (*D*) A basal view of an SBEM reconstruction of all of the hair cells in one neuromast shows that early maturity hair cells (asterisks) but not late maturity or full maturity hair cells bear projections. The plane of view is tangential to the larval surface.

Time-lapse imaging of nascent hair cells during the period of synaptogenesis revealed striking projections that protruded from the base of each soma (Fig. 2A; Supplemental Movies 2,3). Originating 41 min ± 25 min (*N* = 12) after rearrangement and extending up to 15 μm, the projections were variously filamentous, branched, or clavate. The projections extended and retracted dynamically but arose consistently from the same two or three sites on each soma. Fluorescent labeling revealed that the projections contained numerous actin filaments as well as Map1b, indicating the presence of microtubules (Fig. 2B). Projections from nascent hair cells extended toward neighboring mature hair cells in a biased manner: The projections originating from hair cells of each polarity generally approached the vicinity of mature hair cells of the same polarity (Fig. 2C).

The neuromast is a useful model system for mechanosensory epithelia owing to its small size, microscopic accessibility, well-characterized development, and polarity-specific innervation. To ensure that our findings with this preparation are of more general relevance, however, we also conducted time-lapse imaging of the developing inner ear of the zebrafish. Hair cells in the utricle extended from their basal surface projections, whose morphology and behavior matched those of lateral line hair cells (Supplemental Movie 4). For the reasons noted above, we focused our remaining investigations on the projections of lateral line hair cells.

**Figure 3.**
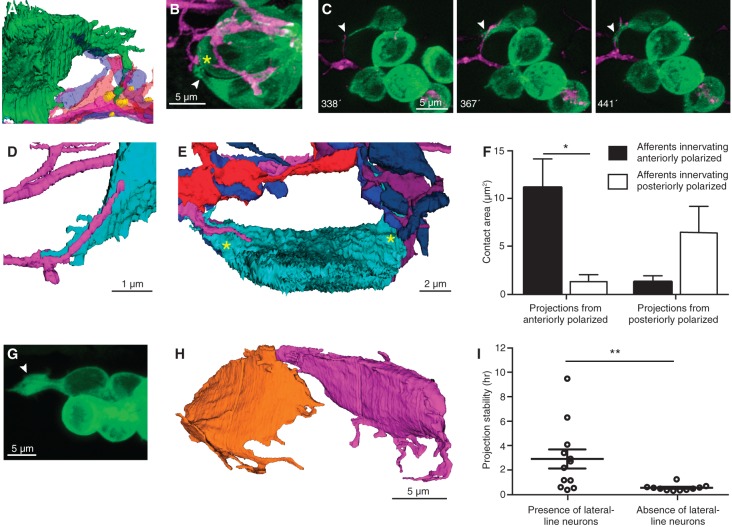
Afferent neurons traverse projections that extend to mature synapses. (*A*) An SBEM reconstruction shows a hair cell projection (green) extending into an aggregation of variously colored axonal terminals beneath mature ribbon synapses made by another hair cell (yellow). (*B*) In a frame from a time-lapse sequence, a projection (arrowhead) extends toward a presumptive ribbon synapse (asterisk). (*C*) A series of time-lapse confocal images shows extension of an afferent terminal along a projection (arrowheads). Times are denoted in minutes after rearrangement. (*D*) A reconstructed afferent terminal (magenta) traverses a hair cell projection (cyan). (*E*) An SBEM reconstruction demonstrates that all neurons contact the nascent cell soma on its two projections (asterisks). (*F*) Projections arising from anteriorly and posteriorly polarized hair cells make the greatest areas of contact with afferent neurons of the same polarity. *P* < 0.02; *N* = 4. (*G*) An unusually large projection (arrowhead) forms in the absence of lateral line neurons. (*H*) The reconstructed sibling hair cells from a larva whose lateral line ganglion had been ablated exhibit numerous and lengthy projections. (*I*) In the absence of lateral line neurons, projections show reduced stability. *P* < 0.01; *N* = 12 and 11.

Intrigued by the appearance of hair cell projections during the period of synapse formation, we investigated more closely the interactions between projections and afferent terminals. The SBEM data, which afforded a view of the projections and their interaction partners on a nanometer scale, confirmed that hair cells extended projections during early maturity but not thereafter (Fig. 2D). No projections were observed in either supporting cells or mantle cells. The projections formed extensive areas of contact with afferent terminals, less contact with efferent fibers, and almost no contact with other hair cells (afferent terminals, 20.0 μm^2^ ± 9.6 μm^2^; efferent terminals, 3.9 μm^2^ ± 3.1 μm^2^; mature hair cells, 0.5 μm^2^ ± 0.5 μm^2^; sibling hair cells, 0.4 μm^2^ ± 0.4 μm^2^; *N* = 4). Although, in time-lapse movies, we frequently observed projections extending to aggregations of afferent terminals beneath mature hair cells, SBEM reconstruction revealed that the projections were prevented from making direct contact with those cells by the intervening nerve fibers (Fig. 3A,B). Exhaustive SBEM reconstruction of other cell types did not reveal any structures that appeared to play a role in the genesis or structure of the projections (Supplemental Movie 5).

The terminal arbors of lateral line afferents include immobile portions that reside beneath mature hair cells as well as thin filopodia that roam throughout the neuromast and initiate contact with nascent hair cells (Faucherre and López-Schier 2014). These filopodia frequently extended along a hair cell projection toward the associated soma (Fig. 3C). The SBEM reconstructions provided several examples of afferent filopodia passing directly along a projection (Fig. 3D). In fact, 94% of the afferent filopodia that colocalized with the somata of nascent hair cells in the time-lapse movies did so at sites from which projections extended (*N* = 77 instances of colocalization at 10 hair cells). The SBEM data revealed that all of the contacts of early maturity hair cells with afferent fibers occurred along projections (Fig. 3E). Moreover, the projections from hair cells of a given polarity contributed significantly greater areas of contact to terminals of the same functional polarity than to those of the opposite polarity (Fig. 3F). This preferential contact occurred even when the projections from hair cells of opposite polarity extended into the same aggregation of afferent neurons (Supplemental Fig. S2).

Because the appearance of projections coincided with the onset of abundant contacts between hair cells and afferent terminals, we wondered whether afferent nerve fibers are necessary for the formation of projections. To address this question, we performed time-lapse imaging of neuromasts in larvae whose afferent neurons had been ablated by ultraviolet irradiation of the lateral line ganglia. SBEM data revealed a complete absence of nerve terminals in the neuromast of a treated specimen and demonstrated that the foramen in the basal lamina through which neurons normally extend from the posterior lateral line nerve into the neuromast was closed. Projections nevertheless arose as usual shortly after the conclusion of cellular rearrangement (Fig. 3G,H). Furthermore, the projections in specimens that underwent ablation were less stable than those in samples that retained afferent terminals (Fig. 3I).

**Figure 4.**
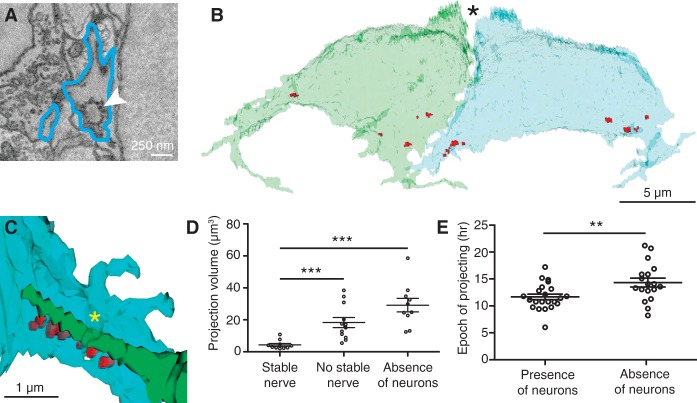
Retraction of projections is associated with stable afferent contact. (*A*) An SBEM image shows an immature synaptic ribbon (arrowhead) within a hair cell projection (blue outline). (*B*) In a reconstruction of the sibling hair cells from a recent division, synaptic ribbons (red) cluster at the sites from which projections emerge. *N* = 10. In this basal view, the partially developed hair bundles are apparent at the cells’ apical ends (asterisk). (*C*) An afferent terminal of the functionally appropriate subpopulation (green) contacts immature synaptic ribbons (red) on a hair cell projection (blue). A sheet of hair cell membrane (asterisk) folds over the afferent terminal. (*D*) Enlarged projections are associated with the absence of stable nerve contacts (*P* < 0.001) or of lateral line neurons (*P* < 0.0001). *N* = 10, 12, and 11. (*E*) The total period during which projections emerge from hair cells increases in the absence of lateral line neurons. *P* < 0.01; *N* = 23 and 18.

In time-lapse images, we often observed afferent terminals halting near the bases of projections. Upon investigating the ultrastructure of such regions by SBEM, we discovered clusters of vesicles as well as immature synaptic ribbons with associated synaptic vesicles, structures nearly absent elsewhere in the cell (Fig. 4A,B). Half of the immature synaptic ribbons (six of 12) were juxtaposed with afferent nerve terminals, which in each case belonged to the subpopulation of appropriate polarity. In two instances, we observed sheets of hair cell membrane embracing the juxtaposed nerve terminals as if they were clamping them in place (Fig. 4C).

The sizes of the projections varied through the course of time-lapse imaging. In hair cells whose bases were stably associated with afferent terminals, the projections were significantly smaller than in those without stably associated afferents (Fig. 4D). Projections also tended to be larger in specimens lacking lateral line neurons (Fig. 4D; Supplemental Movie 6). In the SBEM data, projections associated at their bases with a greater number of unpaired synaptic ribbons had larger volumes. We observed that hair cells in specimens without lateral line neurons continued to extend projections later than those in normal neuromasts (Fig. 4E). Mature hair cells in specimens lacking lateral line neurons nevertheless contained synaptic ribbons and vesicles.

## Discussion

**Figure 5.**
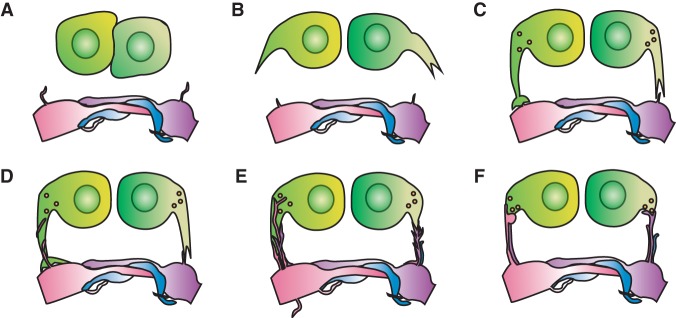
Diagram of a projection-based model of hair cell innervation. (*A*) During their rearrangement, hair cells make few transient contacts and no stable contacts with afferent terminals. (*B*) Following the completion of rearrangement, hair cells develop basal projections. (*C*) The projections extend toward aggregations of afferent neurons at neighboring hair cell synapses. (*D*) Filopodia from afferent terminals extend along the projections toward the hair cell somata. (*E*) Afferent filopodia reach the available presynaptic sites at the bases of the projections. (*F*) Afferent terminals form stable contacts at presynaptic sites, and the hair cell projections retract.

This study used a combination of time-lapse microscopy and SBEM to identify novel cellular projections involved in innervation during the period 2–12 h after hair cell birth. The data suggest the following model of hair cell innervation (Fig. 5). A nascent hair cell extends projections from its soma into the vicinity of mature hair cell synapses nearby. Polarity-appropriate axonal terminals interact with these projections, growing along them to reach synaptic sites on the soma. Finally, after afferent terminals have reached the synaptic sites and become apposed to synaptic ribbons, the hair cell projections retract.

Although the somata of hair cells are generally regarded as stable structures, the present evidence indicates that they participate actively in synaptogenesis. The involvement of hair cell projections as transient scaffolds that mediate polarity-specific innervation at new synaptic sites might offer several advantages. First, the length of the projections makes them large targets for attracting contacts from afferent filopodia. Next, the presence of projections only when prospective synaptic sites are available economizes the process of innervation. Third, their linear structure provides a suitable substrate for directing afferent terminals, which fasciculate extensively with other neurites, to their targets. Finally, the extension of projections toward nearby afferent terminals reduces a terminal's search for new synaptic sites from three dimensions to only one.

Because polarity-specific innervation of lateral line hair cells occurs in the absence of hair cell activity, a chemoaffinity mechanism may be responsible for partner matching (Nagiel et al. 2009). Although hair cell projections are more likely to extend into the vicinity of mature hair cells of the same polarity, this predilection alone is unlikely to confer specific innervation inasmuch as afferent terminals of both functional polarities reside beneath mature hair cells. The chemoaffinity mechanism may instead be based in part on the preferential association of hair cell projections with afferent terminals of the same functional polarity.

It remains to be determined whether projections mediate innervation by the homologous hair cells of the mammalian inner ear as well. Protuberances containing ribbon synapses extend from the bases of type II hair cells in the murine utricle but, unlike the cellular projections reported here, persist through life (Pujol et al. 2014). The transient processes described here resemble the filopodia that establish morphogen gradients and exchange signaling molecules with distant targets (Ramirez-Weber and Kornberg 1999; Hsiung et al. 2005; Sanders et al. 2013; Kornberg and Roy 2014; Roy et al. 2014). Hair cell projections may likewise bear proteins that mediate their interactions with nerve terminals in order to achieve polarity-specific innervation.

## Materials and methods

### Animal care and breeding

Experiments were conducted in accordance with the standards of Rockefeller University's Institutional Animal Care and Use Committee. Zebrafish were maintained under standard conditions (Westerfield 2000). Embryos were raised at 28°C in E3 medium (5 mM NaCl, 0.17 mM KCl, 0.33 mM CaCl_2_, 0.33 mM MgSO_4_) containing 1 μg/mL methylene blue. All experiments used larvae at 2–4 dpf.

The transgenic zebrafish lines included *ET4*, *Tg(pvalb3*:*Ribeye-mCherry)*, *Tg(myo6b*:*actin-GFP)*, *Tg(myo6b*:*Map1b-GFP)*, *Tg(HuC*:*GFP)*, and *Tg(hspGFF4A*;*UAS:nfsb-mCherry)* (Faucherre et al. 2009; Kawakami et al. 2010; West and McDermott 2011; Kindt et al. 2012).

### Classification of hair cells and afferent terminals

The stage of differentiation of each hair cell was determined on the basis of morphological characteristics of the soma, apical surface, and hair bundle; fluorescence expression; and observable cellular behavior (Kindt et al. 2012). A rearranging hair cell is tightly apposed to its sibling cell and occasionally changes position with it, fluoresces dimly in *Tg(myo6b*:*actin-GFP)* larvae, and lacks basal projections and a discernible kinocilium. An early maturity hair cell may display anterior–posterior elongation of the soma, expresses an intermediate level of fluorescence in *Tg(myo6b*:*actin-GFP)* larvae, possesses basal projections, and has a small apical surface with a centrally situated kinocilium. A late maturity hair cell has a soma of intermediate size, fluoresces strongly in *Tg(myo6b*:*actin-GFP)* larvae, may retain regressed basal projections, and possesses a small apical surface with a polarized kinocilium. Finally, a mature hair cell has a rotund soma with a convex base and a large apical surface with a strongly polarized kinocilium. Additional characteristics of hair-bundle morphology have been described at various stages (Kindt et al. 2012).

### Live imaging of larvae

A larva at 2–4 dpf was anesthetized in 600 μM 3-aminobenzoic acid ethyl ester methanesulfonate in E3 medium and mounted in a 35-mm glass-bottomed dish in 1% low-melting-point agarose. Laser-scanning confocal imaging was performed with an inverted Zeiss Axio Observer Z1 with an LSM 780 system equipped with a 60× oil immersion objective lens. Neuromasts were imaged at 20-min intervals as Z-stacks acquired with 0.7-μm steps under laser excitation at 488 nm and 561 nm. Images were deconvolved with AutoQuant X3 software (Media Cybernetics), processed into maximum intensity Z-projections or movies, and analyzed with FIJI (National Institutes of Health). Imaging of the utricule in the inner ear was performed identically to that of neuromasts. Spinning-disk confocal imaging was conducted on an inverted Zeiss Axiovert 200 with a PerkinElmer scanning unit and 63× water immersion objective lens. Neuromasts were imaged as above under laser excitation at 491 nm and 561 nm. Images were processed and analyzed using FIJI. Each image depicts a plane of view tangential to the animal's surface and is oriented with anterior to the left and dorsal upward.

### Image quantification

Contact between afferent terminals and nascent hair cells was judged by their colocalization in image stacks acquired by spinning-disc confocal microscopy. Ribeye and MAGUK puncta were segmented manually, and their areas were measured in FIJI. A projection was considered stable if, between successive time points, it did not retract >50% of its length or shift in azimuthal position by >30° as viewed along the apicobasal axis. A projection was considered for analysis only if it was clearly distinguishable from neighboring GFP-positive hair cells. As a result, approximately one-third of the projections analyzed by confocal microscopy extended toward the center of a neuromast, whereas two-thirds extended toward the periphery. The epoch of projection was considered complete when the hair cell no longer extended projections exceeding 1 µm in length.

The contact areas of projections were calculated by multiplying the contact lengths measured through SBEM by the sectioning interval. Hair cell projections were segmented and measured in deconvolved image stacks imported into Imaris (Bitplane). Each projection was measured beginning at the dilatation of the soma that departed from the smooth contour of the cell membrane. A technician with no knowledge of the sample's identity performed the segmentation.

### Ablation of posterior lateral line ganglia

A zebrafish of the *Tg(hspGFF4A*;*UAS:nfsb-mCherry*;*myo6b*:*actin-GFP)* line was anesthetized at 2 dpf in 600 μM 3-aminobenzoic acid ethyl ester methanesulfonate in E3 medium and mounted in a 35-mm glass-bottomed dish in 1% low-melting-point agarose. The red-fluorescent posterior lateral line ganglion was identified in a Nikon Eclipse Ti inverted wide-field fluorescence microscope with a Micropoint laser ablation system (Andor). Five high-powered shots from a coumarin 440 dye laser obliterated the ganglion. The absence of mCherry-fluorescent neurons was confirmed immediately after laser firing and 24 h later. Efferent neurons, which pass through the ganglion, were assumed to have been ablated as well, a supposition that was confirmed by fluorescence microscopy in *Tg(HuC*:*GFP)* zebrafish and by SBEM.

### Antibody labeling

For antibody labeling, 3.5-dpf *Tg(myo6b*:*actin-GFP)* larvae were fixed overnight at 4°C in 4% aqueous formaldehyde and washed in phosphate-buffered saline solution with 0.1% Tween-20 (PBST). Nonspecific labeling was blocked for 1 h at room temperature with 2 mg/mL bovine serum albumin in PBST before exposure to the primary antibody overnight at 4°C. After a wash, an AlexaFluor 568 anti-mouse secondary antibody was applied for 4 h at room temperature. Pan-MAGUK antibody (NeuroMab) was used at a dilution of 1:500, mouse anti-acetylated tubulin (Sigma-Aldrich) was used at 1:500, and AlexaFluor 568-conjugated anti-mouse secondary antibody (Life Technologies) was used at 1:500. Imaging was performed on an Olympus IX81 confocal microscope with a Fluoview FV1000 laser-scanning system.

### Tissue preparation for EM

SBEM of an entire larval neuromast requires that sectioning commence at one margin of the structure and continue through its complete extent of ∼40 µm. The data acquisition procedure is time-consuming and expensive, however, so it is useful to have a means of restricting the sectioning as much as possible.

As it progresses from behind the otic vesicle to the tail, the primary lateral line primordium of the larval zebrafish typically deposits seven neuromasts on each side of the body. Our studies generally involved the third through sixth of these receptor organs, which could readily be identified and studied at the level of compound and confocal microscopy. For all but one of the SBEM specimens, the neuromast of interest was first imaged for 4–28 h by time-lapse fluorescence confocal microscopy. Within minutes after the conclusion of imaging of a 4-dpf *Tg(myo6b*:*actin-GFP)* larva, we removed the animal from 1% low-melting-point agarose and anesthetized it in a 600-μM solution of 3-aminobenzoic acid ethyl ester methanesulfonate. The fish was fixed for 18 h at 4°C in 200 mM glutaraldehyde, 400 mM formaldehyde, 75 mM sodium cacodylate, 10 mM sucrose, and 1 mM CaCl_2_. To facilitate the subsequent localization of the cells of interest, we photographed the preparation at a magnification great enough to reveal the relevant neuromast but low enough to include several myomeres and landmarks such as melanocytes. The tail was cut transversely 200 μm posterior to the target neuromast and washed in 75 mM sodium cacodylate, 10 mM sucrose, and 1 mM CaCl_2_.

To enhance the contrast of the specimen, it was post-fixed for 1.5 h at 4°C in 80 mM OsO_4_, 75 mM sodium cacodylate, 10 mM sucrose, 35 mM K^+^ ferrocyanide, and 1 mM CaCl_2_. Following a wash in distilled water and treatment for 30 min at 60°C with 100 mM thiocarbohydrazide, the specimen was again washed in distilled water and treated with 80 mM OsO_4_ for 1 h at room temperature. After the sample had been rinsed in distilled water, it was incubated for 16 h at 4°C in 25 mM uranyl acetate. Washes in distilled water were followed by treatment for 30 min at 60°C in 20 mM lead (II) nitrate dissolved in 30 mM L-aspartic acid adjusted to pH 3.5 with 25 mM KOH. After washing, the specimen was dehydrated in a graded series of ethanol concentrations culminating in immersion in 100% ethanol twice for 45 min at room temperature. The sample was then transferred to propylene oxide twice for 45 min.

Each specimen was placed for 24 h in an isovolumetric mixture of propylene oxide and embedding plastic and then transferred to pure plastic mixture and gently stirred for 24 h before being placed into a 2-mm layer of plastic and cured in a vacuum oven for 48 h at 60°C. The embedded sample was trimmed and glued onto a stub for sectioning. Owing to the flat surface of the preparation, it was readily possible to image an embedded specimen. Because of the intense metal deposition, however, transillumination was not possible. It was therefore necessary to locate the appropriate neuromast by use of landmarks such as the ends of fins prior to the heavy metal treatments. The specimen was transected two or three myomeres caudal to the relevant neuromast and secured at its rostral end with epoxy glue to an epoxy specimen capsule held in a microtome chuck.

We next sectioned the face of the mounted plastic block at 1-µm intervals and stained the semithin sections with ethanolic toluidine blue and basic fuchsin. Three techniques helped to determine when sectioning had proceeded to the point at which SBEM should commence. First, cellular landmarks such as melanocytes were apparent in sections, and their positions and shapes could be compared with the images of live or fixed specimens before metal impregnation. Next, owing to the volcano-like shape of a neuromast, it was sometimes possible to see a slight bulging of the skin as the target was neared. Finally and most importantly, the chevrons of successive myomeres provided a quantitative metric of the progress of sectioning.

In most semithin transverse sections, two myomeres are apparent on each side of the body. Because the myomeres are arranged in a forward-pointing arrowhead configuration, sectioning in a caudal-to-rostral direction first encounters the barbed ends of each myomere at the dorsal and ventral larval margins. As sectioning progresses, the cross-sectional area of that myomere grows as its boundaries extend toward the horizontal midline. The adjacent but more caudal myomere meanwhile displays a complementary decrease in area, and, at some level, the pointed end of that myomere shrinks to nothing along the horizontal midline. At that point, the more rostral myomere reaches its maximal cross-sectional area, and the first traces of the next more rostral myomere appear at the dorsal and ventral edges of the specimen. As sectioning continues, the sequence commences anew.

We made use of the progression of myomere boundaries by noting in the live and aldehyde-fixed specimen at what level the caudal edge of a neuromast lay with respect to the underlying myomeres. Semithin sectioning could then continue until the cross-sectional profile of the myomere posterior to the neuromast had reached the appropriate size, halting ∼5 μm posterior to the target neuromast. Finally, the block face was trimmed to ∼200 μm × 200 μm to exclude extraneous tissue and submitted for SBEM.

### SBEM

Four initial samples embedded in Embed-812 were imaged by Renovo Neural, Inc., on a Zeiss Sigma VP scanning electron microscope with a Gatan 3View high-precision ultramicrotome. As the ultramicrotome cut the block face at 50–60 nm increments, images were acquired in high-vacuum mode with a lateral resolution of 5 nm × 5 nm per pixel. Overnight imaging resulting in 700–1000 consecutive images of the tissue sample, which were aligned using the SIFT algorithm of FIJI. Five additional samples embedded in Durcapan were imaged at The Rockefeller University Electron Microscopy Resource Center with a Zeiss Merlin high-vacuum scanning electron microscope with a Gatan 3View2XP camera. The ultramicrotome cut the block face at 30-nm increments with a lateral resolution of 6 nm × 6 nm per pixel. Alignment was performed with Digital Micrograph software.

One data set that included a nearly complete neuromast was chosen for complete reconstruction of the cell membranes. Data annotation technicians were identified through Craigslist and passed a work sample test before being hired. Each cell in the neuromast was given a unique identification number, and the annotator responsible was provided a set of starting coordinates. The cell was reconstructed on the user's computer by outlining its plasmalemma across serial sections with the free software Reconstruct (Fiala 2004). Challenging areas for annotation were noted and evaluated by E. Dow, leaving no areas of significant ambiguity. Cell contours were compiled into a master file that was twice checked independently for accuracy. Surfaces of the cells were rendered from contours in Reconstruct, after which additional rendering and post-production were performed in MeshLab and Blender version 2.72b (https://www.blender.org). Volume and distance measurements were performed in Reconstruct, and measurements of contact areas were conducted in FIJI.

### Statistical analysis

The significance of pairwise differences between groups of samples was computed by Student's two-tailed *t-*tests. Significance is denoted in the graphs as follows: *P* < 0.001 (***), *P* < 0.01 (**), and *P* < 0.05 (*).

## Supplementary Material

Supplemental Material
