# Supplementary material for: Cellular projections from sensory hair cells form polarity-specific scaffolds during synaptogenesis
Source: Genes Dev. 2015 May 15;29(10):1087–94. doi: 10.1101/gad.259838.115 (PMC4441055; doi:10.1101/gad.259838.115)
Supplement: Supplemental Material [file supp_29_10_1087__index.html]

Supplemental Material 

# Cellular projections from sensory hair cells form polarity-specific scaffolds during synaptogenesis

## Supplemental Material

**Files in this Data Supplement:**

- Supp Legends.pdf
- Supp Fig S1.eps
- Supp Fig S2.eps
- Supp Video 1.mov
- Supp Video 2.mov
- Supp Video 3.mov
- Supp Video 4.mov
- Supp Video 5.mov
- Supp Video 6.mov
