# Supplementary material for: Cellular projections from sensory hair cells form polarity-specific scaffolds during synaptogenesis
Source: Genes Dev. 2015 May 15;29(10):1087–94. doi: 10.1101/gad.259838.115 (PMC4441055; doi:10.1101/gad.259838.115)
Supplement: Supplemental Material [file supp_29.10.1087_SuppLegends.pdf]

Supplementary Information

for

Cellular projections from sensory hair cells form polarity-specific scaffolds during synaptogenesis

Eliot Dow, Kimberly Siletti, and A. J. Hudspeth

Supplemental figure legends

Figure S1. Image of a neuromast acquired by serial blockface electron microscopy. Representative cells of the principal types have been outlined. The dark areas at the top of the image represent regions in which the nonconductive plastic has become electrically charged. The complete series of images of which this is part is shown in Video 5.

Figure S2. The projections from anteriorly polarized (AP) and posteriorly polarized (PP) hair cells insinuate into an aggregation of afferent terminals of both functional polarities: A1 and A2 innervate mature anteriorly polarized hair cells, whereas P1 and P2 innervate mature posteriorly polarized hair cells. Note that the protrusion of each polarity of hair cell makes direct contact predominantly with afferent terminals of the polarity-appropriate subpopulation. The profiles labeled E represent the terminal branches of an efferent fiber. The contrast of the micrograph has been reduced to emphasize the traced cellular boundaries.

Video 1. In an SBEM data stack for a developing neuromast, four hundred consecutive images reveal the structure of the neuromast, the assemblage of cells on the right side of the field of view lying above the horizontal grey line representing the basal lamina. Each hair cell is surmounted by a hair bundle comprising a single long, stout kinocilium and a cluster of diminutive stereocilia. Note that axon terminals from the lateral-line nerve penetrate the basal lamina and extend into the neuromast.

Video 2. Hair cells form projections shortly after rearrangement. Timelapse imaging by fluorescence confocal microscopy shows that the two nascent hair cells (green) at the lower edge of the cell cluster extend projections shortly after the completion of their rearrangement. The projections interact with filopodia extending from the afferent terminals (magenta). The two older pairs of hair cells in the middle and top of the neuromast lack projections. This segment represents 8 hr of development.

Video 3. Hair-cell projections persist until contact is made with afferent terminals. The nascent hair cell (green) at the lower left of the cell cluster initially extends large projections in the absence of stable contacts with afferent terminals (magenta). This segment represents 7.5 hr of development.

Video 4. Projections arise from nascent hair cells of the zebrafish inner ear. Timelapse imaging by fluorescence confocal microscopy of the utricular macula of the inner ear reveals the formation of projections in nascent hair cells (green). This segment represents 7.5 hr of development.

Video 5. In a three-dimensional reconstruction of the neuromast from the SBEM data shown in Video 1, cellular membranes were contoured for every cell in and around a neuromast including eight superficial periderm cells (blue), 40 mantle and supporting cells (red), ten hair cells with lengthy kinocilia (green), eight afferent neurons (shades of pink and purple), and one efferent neuron (yellow).

Video 6. Hair cell projections arise in the absence of lateral-line neurons. In a neuromast lacking lateral-line neurons, the two nascent hair cells (green) at the top edge of the cell cluster extend large projections following their rearrangement. This segment represents 6.3 hr of development.
